# A Retrospective Study About the Effectiveness of Anastomosis With a Polyglycolic Acid Sheet in Colorectal Cancer

**DOI:** 10.7759/cureus.56415

**Published:** 2024-03-18

**Authors:** Shoryu Takayama, Keisuke Tomoda, Ken Ishikawa, Masaki Sakamoto, Takeshi Hasegawa, Takehiko Eguchi, Satoru Takayama, Takahiro Mase

**Affiliations:** 1 Surgery, Haibara General Hospital, Shizuoka, JPN; 2 Surgery, Nagoya Tokushukai General Hospital, Kasugai, JPN; 3 Surgery, Oogaki Tokushukai Hospital, Oogaki, JPN

**Keywords:** functional end to end anastomosis, overlap method, double stapling technique, gastroenterological surgery, colorectal cancer, anastomotic leakage, polyglycolic acid sheet

## Abstract

Introduction

Anastomotic leakage is a serious complication in colon and rectal cancer surgeries, contributing to increased mortality rates and extended hospital stays. Despite various preventive measures, including intraoperative assessments and transanal drains, the incidence of anastomotic leakage remains a significant concern. This study investigates the potential efficacy of polyglycolic acid (PGA) sheets in reducing anastomotic leakage rates in gastrointestinal surgeries.

Materials & methods

A retrospective cohort study was conducted between January 2021 and January 2023 at Nagoya Tokushukai General Hospital, Ogaki Tokushukai Hospital, and Haibara General Hospital. A total of 239 patients undergoing colon or rectal cancer surgery were included. Anastomoses were performed with or without PGA sheets, and groups were compared using statistical analyses, including t-tests, Mann-Whitney U tests, and chi-square tests. The primary endpoint was the incidence of anastomotic leakage.

Results

Of the 239 patients, anastomotic leakage occurred in 14 (6%). The PGA use group (52 patients) showed no instances of anastomotic leakage while the PGA non-use group (187 patients) had 14 cases. Comparisons revealed significant differences in anastomotic leakage rates (p=0.04) between the two groups. Univariate analysis demonstrated a lower incidence of anastomotic leakage associated with PGA use (p=0.04). However, no significant differences were observed for transanal drainage (p=0.66), smoking (p=0.76), steroid use (p=1), and preoperative chemotherapy (p=0.07).

Conclusion

This study suggests that the use of PGA sheets in gastrointestinal anastomosis may contribute to a lower incidence of anastomotic leakage. The findings highlight the need for further prospective studies with a larger sample size, distinguishing between colon and rectum surgeries. Despite the limitations of this retrospective study, the observed reduction in anastomotic leakage frequency with PGA sheet use is noteworthy, emphasizing the potential significance of this approach in preventing a critical complication in colorectal surgeries.

## Introduction

Anastomotic leakage is a fatal complication. The occurrence of anastomotic leakage also leads to longer hospital stays. Medical costs also increase. The mortality rate due to anastomotic leakage in colon and rectal cancer surgeries combined has been reported to be 4-18.6% [1-4], and the length of hospital stay increases 3-4 times when anastomotic leakage occurs compared to no anastomotic leakage [5].

Various measures have been taken to prevent anastomotic leakage, including intraoperative blood flow assessment [6] and postoperative placement of a transanal drain [7]. The use of polyglycolic acid (PGA) sheets has been shown to decrease the recurrence rate after pneumothorax surgery [8], and PGA sheets may be effective in tissue reinforcement. Animal studies have suggested that anastomosis with PGA sheets improves the frequency of anastomotic leakage [9]. There is also a report suggesting that PGA sheets in double stapling technique (DST) anastomosis for rectal cancer may be effective for safe reconstruction [10]. However, there is no evidence that PGA sheets improve the anastomotic leakage rate in colorectal cancer surgery.

This study was designed to investigate whether gastrointestinal anastomosis using PGA sheets contributes to the reduction of anastomotic leakage rate.

## Materials and methods

This study is a retrospective cohort study. Patients who underwent surgery for colon and rectal cancer between January 2021 and January 2023 at Nagoya Tokushukai General Hospital, Oogaki Tokushukai Hospital, and Haibara General Hospital will be included. Prior to the study, the Joint Ethical Review Committee reviewed the content of the research protocol from the viewpoints of ethical, scientific, and medical appropriateness, as well as the eligibility of the principal investigators. The study was conducted after the Joint Ethical Review Committee approved the implementation of the study (approval number: TGE02158-016). Informed consent prior to the study was provided on a disclosure and opt-out basis.

Eligible patients were enrolled as research subjects, and information about patients' background, medical history, surgical details, postoperative blood test values, presence of early postoperative complications, including anastomotic leakage, and duration of postoperative hospitalization were corrected. The primary endpoint was the incidence of anastomotic leakage. For the colon, an automated anastomosis machine was used; anastomosis was performed using either the functional end-to-end anastomosis (FEEA) or the overlap method. When PGA sheets were used for colon anastomosis, Tri-Staple™ 2.0 Linforce Reload 60 mm Purple was used; when PGA sheets were not used, Tri-Staple™ 2.0 Curved Tip 60 mm Purple was used. The double-stapling technique was used for anastomosis of the rectum. A Tri-Staple™ EEA™ circular was used, and if a PGA sheet was used, a Neoveil sheet was used. For the use of sheets for anastomosis, we referred to previously reported forms [10].

The PGA sheet use and non-use groups were compared. The anastomotic leakage occurrence group and the non-anastomotic leakage group were also compared. The medians and interquartile ranges (IQR) were calculated for the continuous variables and compared using t-tests. Categorical variables were presented as percentages, and Fisher's exact test was used for the comparison of categorical variables. Differences with P <0.05 were considered statistically significant. Statistical analysis was performed using EZR software [11].

## Results

**Figure 1:**
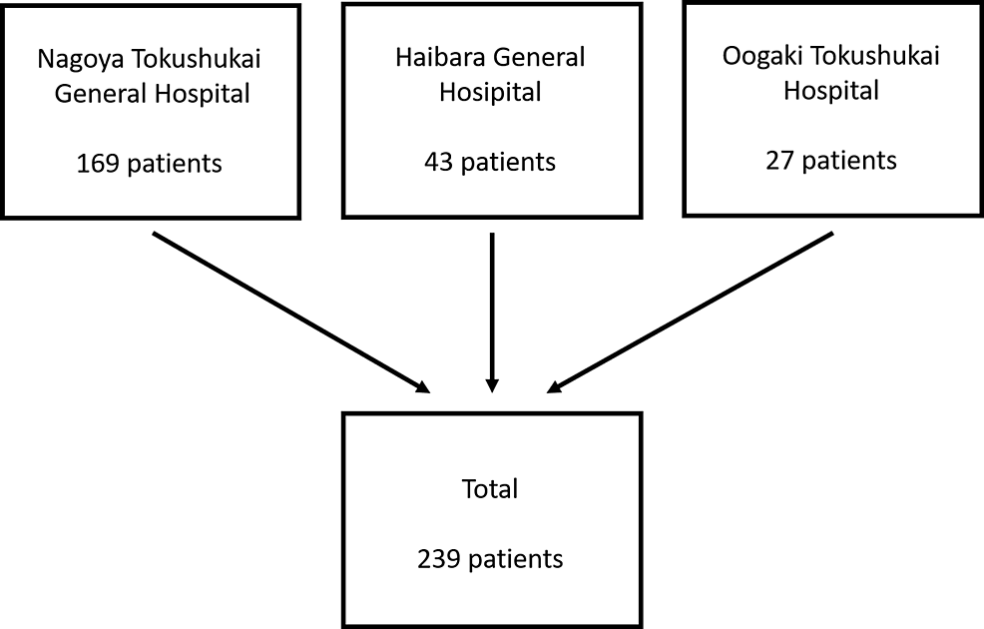
Eligible patients In this study, three hospitals were enrolled. A total of 239 patients were enrolled in the study and all patient information was extracted. The presence of anastomotic leakage and the use of PGA sheets were determined in all patients. PGA, polyglycolic acid

Patients from Nagoya Tokushukai General Hospital, Ogaki Tokushukai Hospital, and Haibara General Hospital were used in this study; patients who underwent surgery for colon or rectal cancer from January 2021 to January 2023 were included. There were 239 patients included in the study. Information was extracted from the hospital's electronic medical records and data for the 239 patients were used without missing data (Figure 1). The male/female ratio was 151 males and 88 females with a mean age of 73 years and a mean BMI of 21.6. Anastomotic leakage occurred in 14 patients (6%). Five cases were colonic, and 9 cases were rectal.

In assessing the potential bias associated with the use of PGA, a comprehensive analysis of various factors was conducted. The data, comprising 187 cases in the PGA- group and 52 cases in the PGA+ group, aimed to investigate whether statistical significance existed across key variables (Table 1) (Table 2).

**Table 1:** PGA-using and non-using groups (t-test) Groups were compared separately by the use of PGA sheets. P-values less than 0.05 were considered statistically significant. The medians and interquartile ranges (IQR) were calculated for the continuous variables and compared using t-tests. PGA, polyglycolic acid; BMI, body mass index; ASA, American Society of Anesthesiologists; PNI, Prognostic Nutritional Index; Alb, albumin; Hb, hemoglobin

Item	PGA - n=187	PGA + n=52	p
Age	74.1	73.2	0.617
BMI	21.6	21.7	0.919
ASA	2.2	2	0.04
Preoperative PNI	38	40	0.142
Length of hospital stay	25.4	17.7	0.054
Preoperative Alb	3.6	3.5	0.644
Preoperative Hb	11.8	12.1	0.569

**Table 2:** PGA-using and non-using groups (Fisher's exact test) Groups were compared separately by the use of PGA sheets. P-values less than 0.05 were considered statistically significant. Categorical variables were presented as percentages, and Fisher's exact test was used for the comparison of categorical variables. PGA, polyglycolic acid

Item	PGA - n=187	PGA + n=52	p
Male, n (%)	116 (62)	35 (67)	0.52
Peritoneal drain +, n (%)	172 (91)	44 (85)	0.117
Transanal drain +, n (%)	20 (10)	7 (13)	0.621
Preoperative chemotherapy +, n (%)	16 (8)	2 (4)	0.376
Antiplatelet drugs +, n (%)	28 (15)	5 (10)	0.373
Anticoagulant +, n (%)	13 (7)	5 (10)	0.554
Smoker +, n (%)	45 (24)	17 (32)	0.215
Anastomotic leakage +, n (%)	14 (7)	0 (0)	0.04

In demographic characteristics, no significant differences were observed in age, BMI, and gender distribution between the PGA+ and PGA- groups (p>0.05). In clinical factors, the ASA score exhibited a statistically significant difference, with PGA- patients having a slightly higher score (p=0.04). Preoperative PNI, length of hospital stay, preoperative Alb, and preoperative Hb did not show statistically significant differences (p>0.05). In procedural factors, the use of peritoneal drain, transanal drain, preoperative chemotherapy, antiplatelet drugs, anticoagulant, and smoking status did not demonstrate significant differences between the two groups (p>0.05). In the primary outcome, a statistically significant difference was observed in the incidence of anastomotic leakage, with no cases reported in the PGA+ group compared to 7% in the PGA- group (p=0.04). The comprehensive analysis suggests that, overall, the utilization of PGA did not introduce substantial bias across various demographic and clinical factors.

Next, univariate analysis was performed to assess the association between various factors and the outcome of interest (Table 3).

**Table 3:** Univariate analysis of anastomotic leakage The groups were compared separately according to the presence or absence of anastomotic leakage. The effect of the risk of anastomotic leakage, as found in previous studies, on the patients in this study was examined. P-values less than 0.05 were considered statistically significant. Odds ratios and confidence intervals are presented for each factor, indicating the strength and range of association with the outcome. CI, confidence interval; OR, odds ratio; PGA, polyglycolic acid

Factor	p	OR (95% CI)
Male	0.26	2.219 (0.564-12.73)
Peritoneal drain +	0.37	inf (0.349 -Inf)
Transanal drain +	0.66	1.331 (0.137-6.53)
Preoperative chemotherapy +	0.07	3.784 (0.613 -16.61)
Antiplatelet drugs +	0.41	1.767 (0.299-7.236)
Anticoagulant +	1	0.941 (0.021- 7.037)
smoker +	0.76	1.151 (0.253-4.185)
PGA +	0.04	0 (0-1.047)
Intraoperative blood transfusion +	0.69	0.448 (0.01-3.183)
Preoperative radiotherapy +	1	0 (0-620.7)
Renal failure +	0.34	2.789 (0.056-25.87)
Steroid +	1	0 (0-18.7)

The male gender did not exhibit a statistically significant association with the outcome (p=0.26, odds ratio (OR)=2.219, 95% Confidence Interval (CI): 0.564-12.73). Peritoneal drain did not show a statistically significant association with the outcome, and odds ratio estimation was not feasible due to zero cases in one group (p=0.37, OR=inf, 95% CI: 0.349 -Inf). Preoperative chemotherapy displayed a borderline association with the outcome, suggesting a potential risk factor (p=0.07, OR=3.784, 95% CI: 0.613-16.61). The use of PGA+ was significantly associated with a decreased likelihood of the outcome (p=0.04, OR=0, 95% CI: 0-1.047), indicating a potential protective effect. Intraoperative blood transfusion did not show a statistically significant association with the outcome (p=0.69, OR=0.448, 95% CI: 0.01-3.183). Transanal drain, Antiplatelet drugs, Anticoagulant, Smoker, Preoperative radiotherapy, Renal failure, and Steroid did not demonstrate significant associations with the outcome in the univariate analysis. The univariate analysis highlighted potential associations between several factors and the outcome of interest. Particularly, PGA+ was associated with reduced risk while preoperative chemotherapy showed a borderline association as a potential risk factor. It is imperative to interpret these findings cautiously, considering the limitations of univariate analysis, and further investigations with larger sample sizes and multivariate analyses are warranted to elucidate the independent impact of each factor on the outcome.

## Discussion

Anastomotic leakage in colon and rectal cancer surgery is a fatal complication. Preventing anastomotic leakage is one of the most important issues in gastrointestinal surgery. Intraoperative endoscopy is useful to confirm that the anastomosis is complete [12]. Intraoperative administration of indocyanine green (ICG) to assess intestinal blood flow is useful [6]. The effectiveness of intraoperative vessel sparing to preserve blood flow has also been reported [13-17]. It is also useful to place a transanal drain to prevent postoperative pressure on the anastomosis [7,18]. The risks of anastomotic leakage have also been reported. Various factors have been reported, including gender (male), history of abdominal or pelvic radiation, preoperative chemotherapy, American Society of Anesthesiologists (ASA) score >2, renal failure, preoperative steroid use, perioperative blood transfusion, smoking, and diabetes [1-4,19-21]. Anastomotic leakage, a fatal complication, is still a possible complication, although many preventive measures have been taken.

As shown in this study, anastomosis with PGA sheets improved the frequency of anastomotic leakage. PGA sheets were found to contribute to a lower re-leak rate in pneumothorax surgery [8]. The reduced recurrence rate of pneumothorax suggests that PGA has a tissue-reinforcing effect. Similarly, PGA sheets are used after endoscopic submucosal dissection (ESD) [22], and there have been reports of their use in the colorectal region [9,10] in expectation of the tissue reinforcing effect of PGA sheets. However, there are few reports on the effectiveness of PGA sheets in surgical anastomosis in the area of lower gastrointestinal cancer.

The PGA group showed a lower incidence of anastomotic leakage (P=0.04) and the use of PGA may have contributed to a shorter hospital stay, although this difference was not statistically significant (Table 1) (Table 2). A comparison was also made to determine the factors of anastomotic leakage in the patient groups of this study (Table 3), and a significantly lower incidence of anastomotic leakage was observed in the PGA group (P = 0.04).

This study did not analyze the differentiation of anastomosis methods. There is a possibility of efficacy bias when the colon and rectum are separated. The patient population in this study did not show a significant difference in the incidence of anastomotic leakage at the reported risk. There is a possibility of bias in the sample. The sample needs to be larger to include more patients who are at risk preoperatively. A multivariate analysis should then be performed that takes into account the reported risk of anastomotic leakage. In addition, this is a retrospective study. A randomized controlled trial is needed to examine the efficacy.

## Conclusions

This retrospective study explored the effectiveness of PGA sheets in reducing anastomotic leakage rates in colorectal cancer surgeries. The findings suggest that PGA sheet use may contribute to a lower incidence of anastomotic leakage, highlighting its potential significance in preventing this critical complication. Despite study limitations, including a small sample size and lack of differentiation between colon and rectum surgeries, the results provide valuable insights. Future investigations, possibly through randomized controlled trials, are essential to establish robust evidence on the efficacy of PGA sheets in preventing anastomotic leakage.
